# Comparison of culture-negative and culture-positive sepsis or septic shock: a systematic review and meta-analysis

**DOI:** 10.1186/s13054-021-03592-8

**Published:** 2021-05-08

**Authors:** Yuting Li, Jianxing Guo, Hongmei Yang, Hongxiang Li, Yangyang Shen, Dong Zhang

**Affiliations:** Department of Intensive Care Unit, The First Hospital of Jilin University, Changchun, 130021 Jilin China

**Keywords:** Culture-negative, Culture-positive, Sepsis, Septic shock, Clinical outcomes, Meta-analysis

## Abstract

**Background:**

Mortality and other clinical outcomes between culture-negative and culture-positive septic patients have been documented inconsistently and are very controversial. A systematic review and meta-analysis was performed to compare the clinical outcomes of culture-negative and culture-positive sepsis or septic shock.

**Methods:**

We searched the PubMed, Cochrane and Embase databases for studies from inception to the 1st of January 2021. We included studies involving patients with sepsis or septic shock. All authors reported our primary outcome of all-cause mortality and clearly compared culture-negative versus culture-positive patients with clinically relevant secondary outcomes (ICU length of stay, hospital length of stay, mechanical ventilation requirements, mechanical ventilation duration and renal replacement requirements). Results were expressed as odds ratio (OR) and mean difference (MD) with accompanying 95% confidence interval (CI).

**Results:**

Seven studies including 22,655 patients were included. The primary outcome of this meta-analysis showed that there was no statistically significant difference in the all-cause mortality between two groups (OR = 0.95; 95% CI, 0.88 to 1.01; *P* = 0.12; Chi-^2^ = 30.71; *I*^2^ = 80%). Secondary outcomes demonstrated that there was no statistically significant difference in the ICU length of stay (MD = − 0.19;95% CI, − 0.42 to 0.04; *P* = 0.10;Chi-^2^ = 5.73; *I*^2^ = 48%), mechanical ventilation requirements (OR = 1.02; 95% CI, 0.94 to 1.11; *P* = 0.61; Chi^2^ = 6.32; *I*^2^ = 53%) and renal replacement requirements (OR = 0.82; 95% CI, 0.67 to 1.01; *P* = 0.06; Chi-^2^ = 1.21; *I*^2^ = 0%) between two groups. The hospital length of stay of culture-positive group was longer than that of the culture-negative group (MD = − 3.48;95% CI, − 4.34 to − 2.63; *P* < 0.00001;Chi-^2^ = 1.03; *I*^2^ = 0%). The mechanical ventilation duration of culture-positive group was longer than that of the culture-negative group (MD = − 0.64;95% CI, − 0.88 to − 0.4; *P* < 0.00001;Chi-^2^ = 4.86; *I*^2^ = 38%).

**Conclusions:**

Culture positivity or negativity was not associated with mortality of sepsis or septic shock patients. Furthermore, culture-positive septic patients had similar ICU length of stay, mechanical ventilation requirements and renal replacement requirements as those culture-negative patients. The hospital length of stay and mechanical ventilation duration of culture-positive septic patients were both longer than that of the culture-negative patients. Further large-scale studies are still required to confirm these results.

## Key messages

- Culture positivity or negativity was not associated with mortality of sepsis or septic shock patients.
- Culture-positive septic patients had similar ICU length of stay, mechanical ventilation requirements and renal replacement requirements as those culture-negative patients.
- The hospital length of stay and mechanical ventilation duration of culture-positive septic patients were both longer than that of the culture-negative patients.

## Introduction

The incidence of sepsis and septic shock has been increasing worldwide over the past decade, and its morbidity and mortality are still unacceptably high [1]. Mortality from sepsis and septic shock remains incredibly high, ranging between 20 and 40%, depending on the severity of illness [2, 3]. Guidelines and protocols for sepsis and septic shock treatment have been published and modified over the past two decades. These guidelines widely recommend empirical application of broad-spectrum antibiotics, and several studies have demonstrated that time to effective antibiotic therapy reduces patient mortality [4].

According to The Third International Consensus Definitions for Sepsis and Septic Shock (Sepsis-3), sepsis was defined as life-threatening organ dysfunction caused by a dysregulated host response to infection. Patients with septic shock can be identified with a clinical construct of sepsis with persisting hypotension requiring vasopressors to maintain mean arterial pressure(MAP) ≥ 65 mm Hg and having a serum lactate level > 2 mmol/L (18 mg/dL) despite adequate volume resuscitation [5]. Sepsis involves a wide array of sources and microorganisms, only a fraction of which are microbiologically documented. Sepsis/septic shock without a microbiologically documented infection is called culture-negative sepsis/septic shock. Determination of culture-negative septic shock was based on the diagnosis of septic shock in the absence of positive pathogen cultures from blood, sputum, body fluids, or other tissues. Previous studies have shown that the proportion of culture-negative cases was between 28 and 49% of all patients with sepsis [6–8]. Culture-negative sepsis poses special diagnostic challenges to both clinicians and microbiologists and further questions the validity of sepsis definitions [9].

Sepsis or septic shock is a highly heterogeneous syndrome, affecting patients with various underlying conditions. The possibility exists that culture-negative cases may differ in fundamental ways from culture-positive sepsis or septic shock with respect to pathophysiology, epidemiology, and treatment responses [10]. Mortality and other clinical outcomes between culture-negative and culture-positive septic patients have been documented inconsistently and are very controversial. Therefore, we conducted a meta-analysis, which extracted results from published cohort studies to compare the clinical outcomes of culture-negative and culture-positive sepsis or septic shock.

## Methods

This systematic review and meta-analysis is reported according to the Preferred Reporting Items for Systematic Reviews and Meta-Analyses (PRISMA) guidelines [11]. Ethical approval was not necessary for this study because it was a review of the published literature.

### Search strategy

**Fig. 1:**
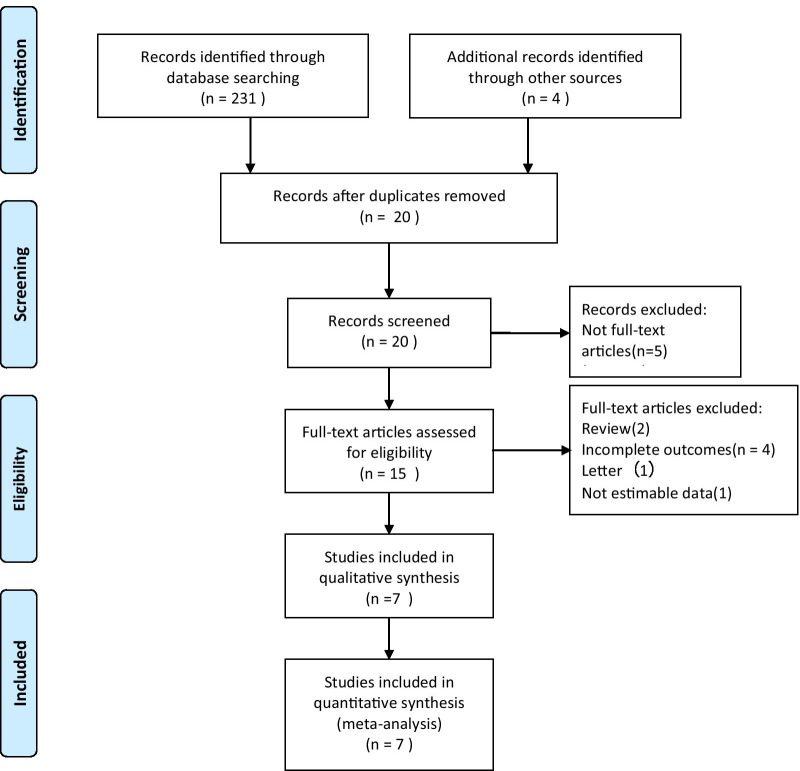
Flowchart of literature selection

We searched the PubMed, Cochrane, and Embase databases for studies from inception to the 1st of January 2021 using the following search terms: culture-positive, culture-negative, sepsis, severe sepsis, septic shock. The search was slightly adjusted according to the requirements of the different databases. The authors’ personal files and reference lists of relevant review articles were also reviewed. The flowchart of the search strategies is summarized in Fig. 1.

### Types of outcome measures

The primary outcome was all-cause mortality, all-cause mortality included hospital mortality, 28-day mortality and 90-day mortality. Secondary outcomes were intensive care unit (ICU) length of stay, hospital length of stay, mechanical ventilation requirements, mechanical ventilation duration and renal replacement requirements. Weighted means were calculated based on the number of patients in each study.

### Study selection

The inclusion criteria were as follows: (1) prospective and retrospective cohort studies; (2) patients with sepsis or septic shock; (3) all authors reported our primary outcome of all-cause mortality; (4) clearly comparing culture-negative versus culture-positive patients with clinically relevant secondary outcomes. We excluded studies including not estimable data [12] and without clear comparisons of the outcomes. In addition, we excluded letter and review.

### Quality assessment

Two reviewers (YL and JG) independently performed quality assessment. The quality of studies was assessed using the Newcastle-Ottawa scale (NOS) for cohort studies [13]. NOS allocates a maximum of 9 points according to the quality of the selection, comparability and outcomes of the cohort study populations. Study quality was defined as poor (0–3), fair (4–6) or good (7–9). The quality of the included cohort studies is presented in Table 1.

**Table 1:** Quality of the included cohort studies (The Newcastle–Ottawa scale)

Study	Selection				Comparability	Outcome			
	Representativeness of the exposed cohort	Selection of the non-exposed cohort	Ascertainment of exposure	Demonstration that outcome of interest was not present at the start of study	Comparability of cohorts on the basis of the design or analysis	Assessment of outcome	Was follow-up long enough for outcomes to occur	Adequacy of follow-up of cohorts	Total score
Phua et al. [16]	☆	☆	☆	☆	☆☆	☆	☆	☆	9
Bast et al. [17]	☆	☆	☆	☆	☆	☆	☆	☆	8
Easaw et al. [18]	☆	☆	☆	☆	☆☆	☆	☆	☆	9
Kethireddy et al. [19]	☆	☆	☆	☆	☆☆	☆	☆	☆	9
Sigakis et al. [20]	☆	☆	☆	☆	☆☆	☆	☆	☆	9
Hazwani et al. [21]	☆	☆	☆	☆	☆	☆	☆	-	7
Kim et al. [22]	☆	☆	☆	☆	☆☆	☆	☆	☆	9

### Statistical analysis

Statistical analyses were performed using Review Manager Version 5.3 (RevMan, The Cochrane Collaboration, Oxford, UK). Odds ratio (OR) with 95% confidence intervals (CIs) was calculated for dichotomous variables. As to the continuous variables, mean difference (MD) and 95% CI were estimated as the effect result. A random-effect model was used to pool studies with significant heterogeneity, as determined by the Chi-squared test (*P* < 0.10) and inconsistency index (*I*^2^ ≥ 50%)[14]. Some of the selected continuous variables were represented by the median (interquartile range). We calculated their mean and standard deviation according to the sample size with an calculator [15] and then performed meta-analysis. A *P* value < 0.05 was set as the threshold of statistical significance.

## Results

### Study characteristics

The search strategy identified 235 studies, and the data were from seven cohort studies comprising 22,655 patients (Table 2) [16–22]. The characteristics of the included studies are shown in Table 2. A total of seven eligible studies were published between 2013 and 2021. Among these studies, two studies were conducted in USA, one study was conducted in Singapore, one study was conducted in Germany, one study was conducted in Saudi Arabia, one study was conducted in Korea, one study was conducted in Canada, USA and Saudi Arabia. Six of these studies were single-center studies, and one was multicenter study. The proportion of patients with culture-positive sepsis or septic shock is about 40.1% (9086/22655). The median percentage of sepsis episodes, which were culture negative, was 49.3%, but there was variability across studies from a minimum of 30.6% to a maximum of 89.4% with an interquartile range of 41.1–52.5%.

**Table 2:** The basic characteristics of studies included in meta-analysis

Author	Year	Country	Study period	All-cause mortality	Study design	No. of patients		
						Total	Culture negative	Culture positive
Phua et al. [16]	2013	Singapore	2004–2009	Hospital	Single-center, prospective cohort study	1001	415	586
Bast et al. [17]	2015	Germany	2009–2014	28-day	Single-center, retrospective cohort study	584	288	296
Easaw et al. [18]	2017	USA	Apr. 2016–Dec. 2016	All-cause	Single-center, retrospective cohort study	80	42	38
Kethireddy et al. [19]	2018	Canada, USA, Saudi Arabia	Jan. 1997–Dec. 2010	Hospital	Multicenter, retrospective cohort study	8670	2651	6019
Sigakis et al. [20]	2019	USA	Jan. 2007–May 2014	Hospital	Single-center, retrospective cohort study	10,393	9288	1105
Hazwani et al. [21]	2020	Saudi Arabia	Apr. 2015–Jan. 2018	All-cause	Single-center, retrospective cohort study	209	179	30
Kim et al. [22]	2021	Korea	Jan. 2014–Dec. 2018	90-day	Single-center, retrospective cohort study	1718	706	1012

### Primary outcome

**Fig. 2:**
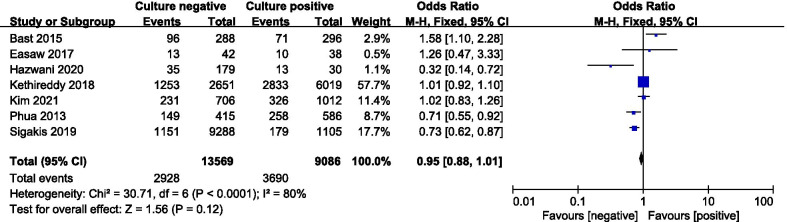
Forest plot for all-cause mortality

**Fig. 3:**
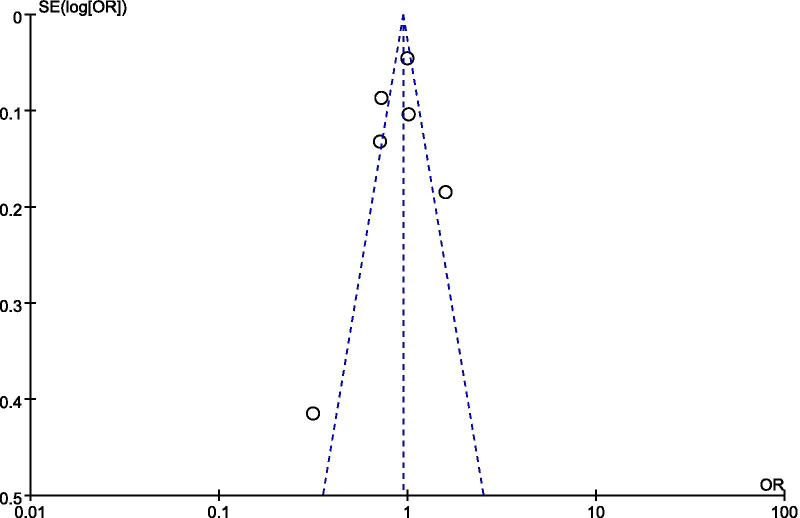
Funnel plot for all-cause mortality

A total of seven studies including 22,655 patients were included, and the all-cause mortality was about 29.2% (2928/13569 in the culture-negative group and 3690/9086 in the culture-positive group). There was no statistically significant difference in the all-cause mortality between two groups (OR = 0.95; 95% CI, 0.88 to 1.01; *P* = 0.12; Chi-^2^ = 30.71; *I*^2^ = 80%) (Fig. 2). A funnel plot was used to assess the publication bias (Fig. 3).

### Secondary outcomes

#### ICU length of stay

**Fig. 4:**
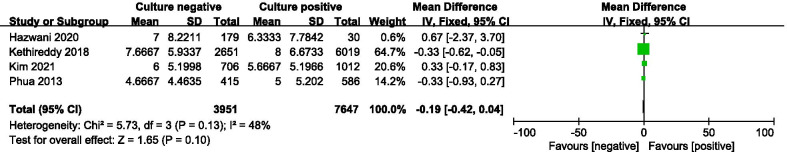
Forest plot for ICU length of stay

Four of included studies were analyzed to assess the ICU length of stay (day). There was no statistically significant difference in the ICU length of stay between two groups (MD = − 0.19;95% CI, − 0.42 to 0.04; *P* = 0.10; Chi^2^ = 5.73; *I*^2^ = 48%) (Fig. 4).

#### Hospital length of stay

**Fig. 5:**
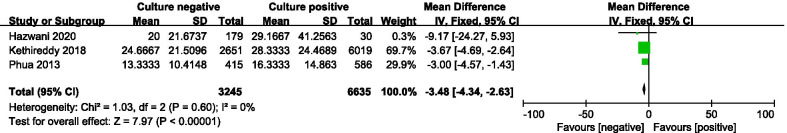
Forest plot for hospital length of stay

Three of included studies were analyzed to assess the hospital length of stay (day). The hospital length of stay of culture-positive group was longer than that of the culture-negative group (MD = − 3.48;95% CI, − 4.34 to − 2.63; *P* < 0.00001; Chi-^2^ = 1.03; *I*^2^ = 0%) (Fig. 5).

#### Mechanical ventilation requirements

**Fig. 6:**
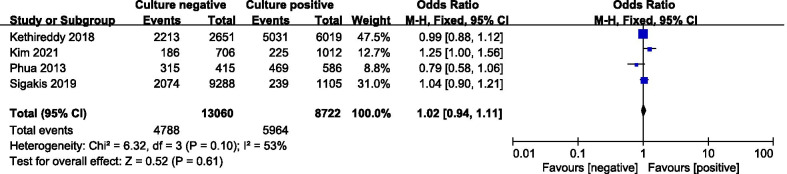
Forest plot for mechanical ventilation requirements

Four of included studies were analyzed to assess the mechanical ventilation requirements. There was no statistically significant difference in the mechanical ventilation requirements between two groups (OR = 1.02; 95% CI, 0.94 to 1.11; *P* = 0.61; Chi^2^ = 6.32; *I*^2^ = 53%) (Fig. 6).

#### Mechanical ventilation duration

**Fig. 7:**
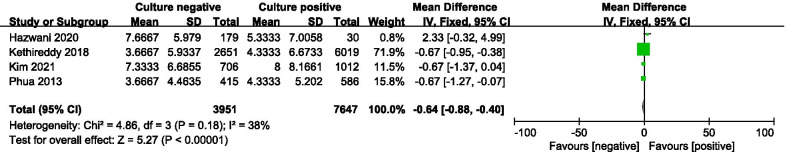
Forest plot for mechanical ventilation duration

Four of included studies were analyzed to assess the mechanical ventilation duration (day). The mechanical ventilation duration of culture-positive group was longer than that of the culture-negative group (MD = − 0.64;95% CI, − 0.88 to − 0.4; *P* < 0.00001;Chi-^2^ = 4.86; *I*^2^ = 38%) (Fig. 7).

#### Renal replacement requirements

**Fig. 8:**
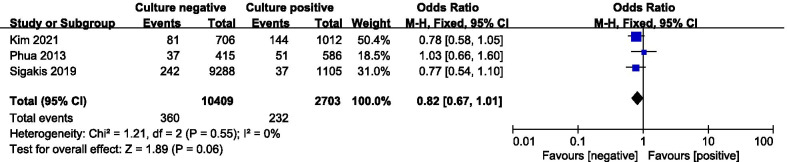
Forest plot for renal replacement requirements

Three of included studies were analyzed to assess the renal replacement requirements. There was no statistically significant difference in the renal replacement requirements between two groups (OR = 0.82; 95% CI, 0.67 to 1.01; *P* = 0.06; Chi-^2^ = 1.21; *I*^2^ = 0%) (Fig. 8).

## Discussion

This systematic review and meta-analysis of seven studies including 22,655 patients compared clinical outcomes of culture-negative and culture-positive sepsis or septic shock patients. We found that only about 40.1% of patients with sepsis or septic shock had a culture-positive infection. Culture-negative and culture-positive patients with sepsis or septic shock demonstrated a similar mortality. The all-cause mortality was about 29.2%, and we did not identify there was statistically significant difference in the all-cause mortality between culture-negative and culture-positive groups. Clinical picture of laboratory values and vital signs had only fair discrimination between culture-negative and culture-positive patients and that culture-negative and culture-positive patients had mostly similar risk factors for death [20].

Why should patients presenting with a clinical syndrome of sepsis or septic shock have culture-negative infection? First, the patients may have been prescribed empirical antibiotics at local clinics before sepsis or septic shock developed [23]. The most important predictor of culture negativity was receipt of antibiotics within the preceding forty-eight hours [20]. Second, the proportion of sepsis or septic shock cases caused by atypical pathogens, including fungal and viral infections, might be increasing [24, 25]. Microbiologically documented infections may include non-culturable pathogens such as viruses, parasites, and probably fungi. It should be acknowledged that viral/fungal/parasite infections might not be so different from culture-positive infections in terms of effective definitive antimicrobial therapy. Conventional microbiological methods frequently not succeed in identifying a microorganism due to various reasons related to technical issues or intrinsic to the microorganism. Promising researches using polymerase chain reaction (PCR) methods showed that microbial deoxyribonucleic acid (DNA)could be rapidly detected in blood of septic patients and could detect potentially significant fungi and bacteria not retrieved from blood culture [26, 27]. In addition, sputum cultures had a quite low positivity rate, but bronchial aspiration could enhance the possibility of identifying the causative pathogens [28]. Third, many patients having culture-negative sepsis or septic shock might actually have non-infectious causes, such as metabolic disorders, tissue injuries, inflammatory diseases, adverse effects of drugs, malignancies and subarachnoid hemorrhage [29, 30].

Besides all-cause mortality, culture-negative and culture-positive patients with sepsis or septic shock demonstrated similar ICU length of stay, mechanical ventilation requirements and renal replacement requirements in our meta-analysis. However, the hospital length of stay and mechanical ventilation duration of culture-positive group were both longer than that of the culture-negative group. These differences are likely due to differences in patient populations, proportions of the sites of infection, and resistance of the bacteria to antibiotics. The longer mechanical ventilation duration and hospital length of stay that we observed in culture-positive patients are likely attributed to the greater occurrence of risk factors, such as older age, higher proportion of malignancies and higher Acute Physiology and Chronic Health Evaluation II (APACHE II) Score. Since sepsis and septic shock are heterogeneous syndromes, the sites of infection were also quite different between the two groups. Previous retrospective studies demonstrated that culture-positive patients with intra-abdominal and lung infections were associated with poor clinical outcomes [31, 32]. However, urinary tract infections were associated with better clinical outcomes than that of the others [33]. We consider that culture negativity might imply susceptibility to the initial antibiotic regimens prescribed, leading to a lesser severity of disease. In addition, the clinical outcomes may be associated with not only the infection sources but also the management of the sepsis and septic shock.

What are the implications of our meta-analysis’s results? The Surviving Sepsis Campaign guidelines recommended early administration of broad-spectrum antibiotics in an effort to improve outcomes in culture-negative or culture-positive sepsis [34]. Every hour of delay in the administration of effective antibiotics from the onset of septic shock will result in increased mortality [35]. Early antimicrobial therapy is deemed appropriate among culture-negative septic patients if they are consistent with national guidelines for the clinical syndrome. Multiplex PCR amplification techniques should be used for the quantification of fungi, viruses and bacteria to elucidate the true-negative and false-negative rates of cultures [36, 37]. If pathogens are not detected, interventional laboratory tests should be used to escalate, continue, narrow or cease antibiotics coupled with a search for non-infectious causes [38].

There are several limitations in our meta-analysis. First, the number of included cohort studies is small. Further large-scale clinical studies should be conducted in order to confirm these results. Second, many of the secondary outcomes such as ICU length of stay, hospital length of stay, mechanical ventilation requirements, mechanical ventilation duration and renal replacement requirements were not included in all of the studies examined in this meta-analysis. Third, there was still substantial heterogeneity among the included cohort studies. Very heterogeneous populations were included in both retrospective and prospective cohort studies. Fourth, suspected source of infection might be the main effect modifiers because some sources of infection have high rates of culture positivity and low rates of mortality (especially urinary tract) and some have low rates of culture positivity and high mortality (especially pulmonary). However, not all of the included studies specified the suspected source of infection. Few of them provided the mortality comparison of culture-negative versus culture-positive infection stratified by source of infection. Fifth, due to the limitation of enrolled studies, our primary outcome all-cause mortality included in-hospital mortality, 28-day mortality and 90-day mortality. As we all understand, these mortality rates are not interchangeable and they are depending on the mortality provided in each included study. Therefore, our results should be interpreted with caution.

## Conclusions

We found that only about 40.1% of patients with sepsis or septic shock had a culture-positive infection. Culture positivity or negativity was not associated with mortality of sepsis or septic shock patients. Early antimicrobial therapy is deemed appropriate among culture-negative septic patients if they are consistent with national guidelines for the clinical syndrome. Furthermore, culture-positive septic patients had similar ICU length of stay, mechanical ventilation requirements and renal replacement requirements as those culture-negative patients. The hospital length of stay and mechanical ventilation duration of culture-positive septic patients were both longer than that of the culture-negative patients. Further large-scale studies are still required to confirm these results.

## Data Availability

All data generated or analyzed during this study are included in this published article.

## Abbreviations

Sepsis-3: The Third International Consensus Definitions for Sepsis and Septic Shock
MAP: Mean arterial pressure
ICU: Intensive care unit
PRISMA: Preferred Reporting Items for Systematic Reviews and Meta-Analyses
NOS: Newcastle–Ottawa scale
OR: Odds ratio
CI: Confidence interval
PCR: Polymerase chain reaction
DNA: Deoxyribonucleic acid
APACHE II: Acute Physiology and Chronic Health Evaluation II
